# Stent Graft Migration Causing Two Aortic Wall Injuries 10 Years After Type II Hybrid Arch Repair

**DOI:** 10.1016/j.atssr.2023.05.008

**Published:** 2023-06-07

**Authors:** David G. Guzzardi, Daniyil A. Svystonyuk, Eric J. Herget, Kenton L. Rommens, R. Scott McClure

**Affiliations:** 1 Division of Vascular Surgery, Department of Surgery, McMaster University, Hamilton, Ontario, Canada; 2 Section of Cardiac Surgery, Department of Cardiac Sciences, Libin Cardiovascular Institute, University of Calgary, Calgary, Alberta, Canada; 3 Division of Interventional Radiology, Department of Diagnostic Imaging, Libin Cardiovascular Institute, University of Calgary, Calgary, Alberta, Canada; 4 Section of Vascular Surgery, Department of Surgery, Libin Cardiovascular Institute, University of Calgary, Calgary, Alberta, Canada

## Abstract

Hybrid arch repair (HAR) combines surgical reconstruction of the ascending aorta and arch debranching with stent graft deployment into the descending thoracic aorta in an effort to reduce the morbidity associated with conventional open total arch replacement. We describe a case of delayed presentation for 2 thoracic aortic wall injuries caused by stent graft migration after type II HAR. This report highlights an important late complication of HAR and the need for careful device selection. It also underlines the importance of lifelong imaging surveillance for patients undergoing complex aortic arch reconstruction.

Hybrid arch repair (HAR) is an alternative consideration to conventional open total arch reconstruction (TAR). Although results are mixed,^1^^,^^2^ combining great vessel debranching with endovascular stent graft deployment may mitigate perioperative morbidity relative to TAR. Still, HAR can incur stent-related issues unique to stent use: endoleak, retrograde type A aortic dissection, stent migration, and stent-induced new entry (SINE) tears. This necessitates anatomic suitability and careful device selection. We present a unique case of 2 delayed complications after type II HAR, in which stent design and stent migration contributed to aortic wall injuries of both the ascending and descending aorta. The aortic injuries were addressed in a staged hybrid approach. Given the widespread use of HAR techniques, practitioners should be aware of this potential complication and its management.

A 69-year-old man first presented in 2010 with an acute DeBakey I aortic dissection. A type II HAR was successfully performed with a 28-mm 4-branched Vascutek Bavaria graft.^3^ Using the right axillary artery and right atrium, cardioplumonary (CPB) and mild hypothermia were instituted (30 °C). A cross-clamp was placed to facilitate replacement of the ascending aorta and released before sequential debranching of the head vessels. Once the patient was weaned from CPB, a Cook Zenith TX2 thoracic stent graft (36 × 202 mm) was deployed antegrade across the arch from zones 0 to 4 (Figure 1).^3^ The patient’s medical history was significant for gout, rheumatoid arthritis, and lupus anticoagulant. The patient did well postoperatively. Incidental note of thrombosis to the left subclavian bypass limb without sequelae was identified at 9 months on routine imaging surveillance.

**Figure 1:**
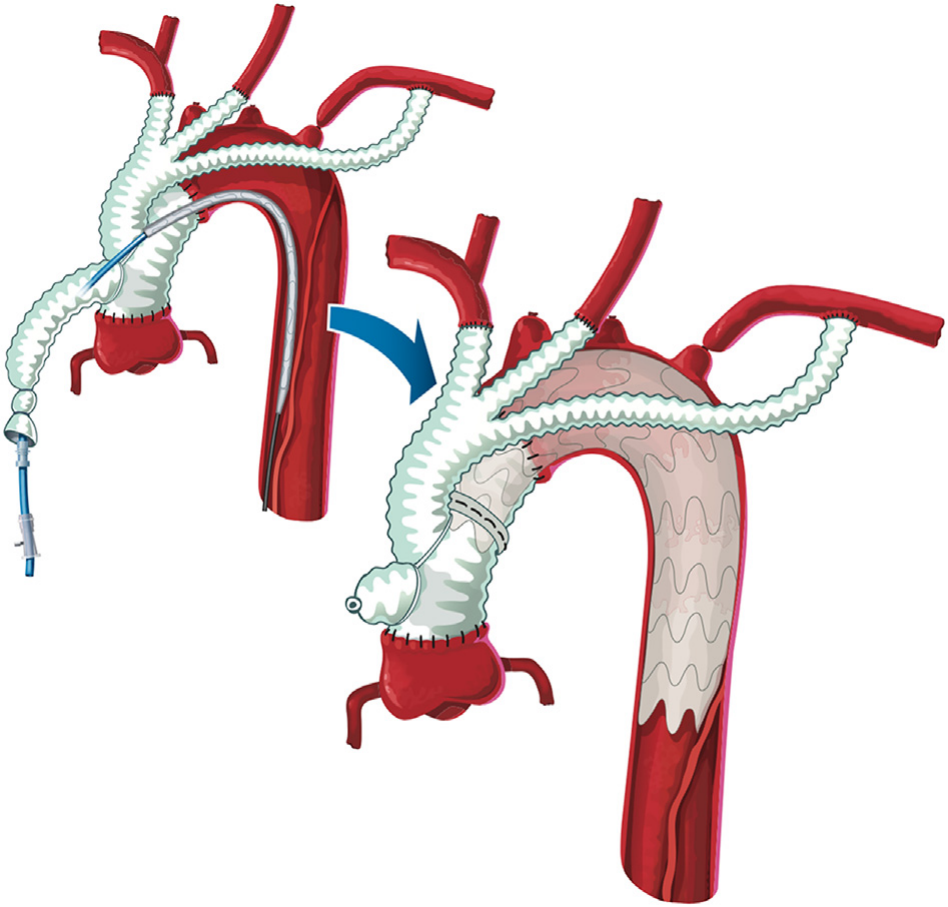
Depiction of the indexed procedure from 2010 (hybrid II arch repair).

Three years after the index operation, a distal SINE tear in zone 4 of the descending aorta was noted on routine computed tomography imaging. The defect was small and the patient was asymptomatic, so surveillance was continued. With stable dimensions over time, computed tomography surveillance was extended to 2-year intervals. Unexpectedly, 10 years after the index surgery (78 years old), a second SINE tear was identified within the ascending aorta at zone 0C. The stent graft had migrated from its original proximal seal zone within the Bavaria graft to injure the aortic wall, causing a 1.2-cm pseudoaneurysm. The wall injury was associated with increased false lumen size within the aortic arch, having pronounced thrombus and a type IA endoleak. The aortic arch increased in diameter to 5.4 cm (previously 4.2 cm). A new type IB endoleak was also present (Figure 2A). The patient remained asymptomatic and felt well.

**Figure 2:**
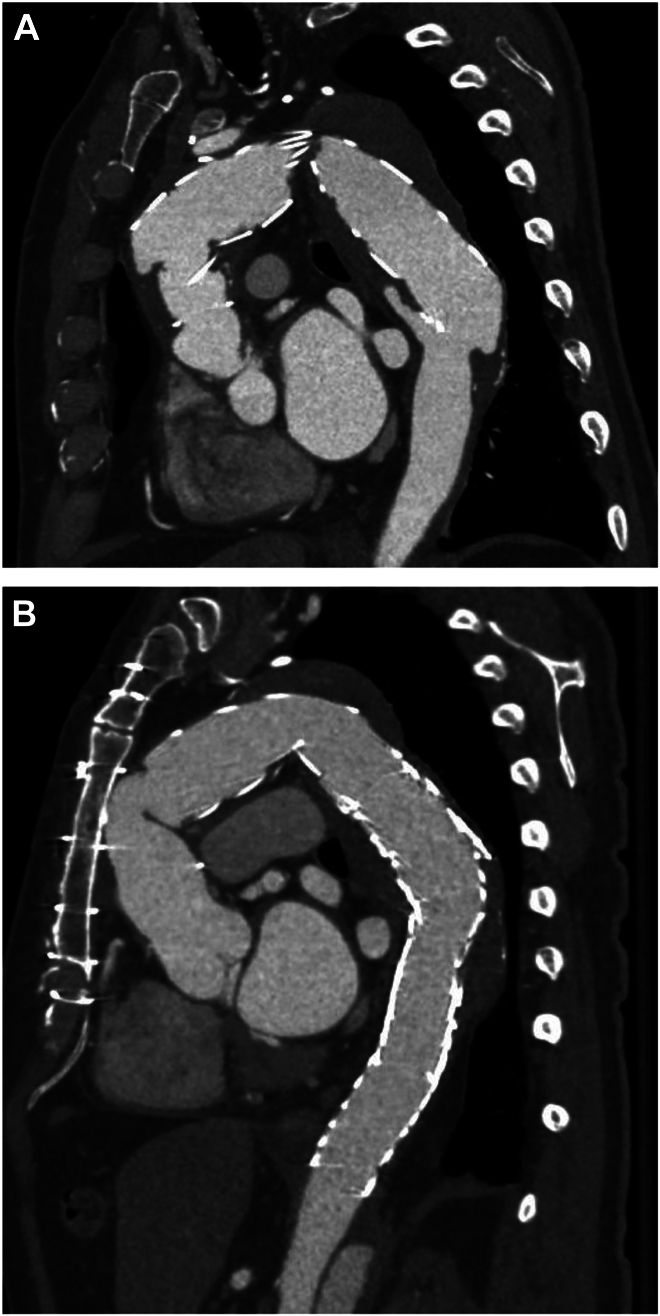
Computed tomography of hybrid arch repair. (A) Interval imaging 10 years after index hybrid arch repair surgery demonstrating proximal blowout and distal stent-induced aortic wall injury. (B) Imaging after definitive ascending aortic repairs.

The patient underwent urgent redo surgical ascending and aortic arch reconstruction, followed by endovascular repair of the descending aorta months later. Given the proximal location of the debranched head vessels (which complicates antegrade cerebral perfusion strategies), after safe sternal reentry, femoral-femoral CPB was instituted. With 7 minutes of pure deep hypothermic circulatory arrest (18 °C) and 20 minutes of adjunct bilateral antegrade cerebral perfusion, a 34-mm Gelweave Ante-Flo graft (Terumo Aortic) was anastomosed to the proximal aspect of the Zenith TX2 stent. The original debranched head vessel limbs were then reimplanted into the graft as an island. Postoperatively, the patient had brief aphasia secondary to a small left middle cerebral artery infarct but had full recovery. His postoperative course was otherwise unremarkable. Eleven months later (delayed because of COVID-19 protocols), the patient underwent a zone 4-5 endovascular extension with 2 GORE cTAG (Gore Medical) active control system stent grafts (31 mm × 10 cm and 37 mm × 15 cm) to exclude the residual type IB endoleak and the chronic distal pseudoaneurysm that had increased to 4.6 cm (Figure 2B). The patient’s postoperative course was remarkable for exacerbation of preexisting idiopathic normal-pressure hydrocephalus managed conservatively, with steady improvement over time.

## Comment

The aortic arch increases the complexity of surgical repair. Although TAR is the "gold standard," HAR with open aortic surgery and thoracic endovascular aortic repair (TEVAR) in combination has emerged as an alternative in select scenarios.^3^ Although long-term outcomes for TAR and TEVAR are separately well delineated,^4^ long-term outcomes for HAR, by contrast, as well as the management of late complications are less understood.^5^ Most studies are limited to small institutional series with a focus on acute or subacute events.

The case herein demonstrates a multifaceted complication after HAR whereby thoracic stent migration caused 2 discrete aortic wall injuries on either side of the aortic arch in a delayed fashion, a decade after the index HAR surgery. Stent-induced wall injury has been reported to occur in up to 9% of TEVAR cases; most injuries have occurred in the setting of an acute dissection, with stents having bare springs or barbs.^6^ Reinterventions were required in one-third of these cases, and they occurred up to 10 years after the index procedure. Higher radial force devices and the degree of oversizing were speculated to contribute to these complications and may provide some insight into the 2 wall injuries reported in this case. Here, the use of a Cook TX2 endograft, designed as a straight conduit for the descending aorta rather than to be curved within the arch, was almost certainly a contributing factor. Migration due to an insufficiently long proximal seal on the outer curvature coupled with straightening of the stent itself likely injured the aortic wall. By contrast, endografts with enhanced arch conformability as well as alternative devices and approaches for arch reconstruction, such as the GORE TBE stent graft or Terumo Aortic RelayBranch system, have since enabled arch debranching while providing a conduit in the descending aorta that may lessen the material property differences between native aorta and stiff protheses. That 2 separate SINE tears required repair in this case presents a unique challenge for surgeons, and use of both open and endovascular strategies to address these complications supports the need for patient-specific tailoring of therapies. Joo and colleagues^7^ in a review of HAR reported that elective repair of HAR complications is preferable to either emergent surgical or conservative management. Such data, in addition to the case we present, highlight the need for ongoing, lifelong surveillance for this population of patients.
